# Vesico Vaginal Fistula

**Published:** 1857-08

**Authors:** 


*Vesico Vaginal Fistula.*—Dr. Bozeman's button-suture, a modification of Dr. Simms clamp-suture, seems to be rapidly gaining favor in the *Surgical World* and will doubtless become the most acceptable mode of operating for this most obstinate affection.

